# Middle manager responses to hospital co-workers’ unprofessional behaviours within the context of a professional accountability culture change program: a qualitative analysis

**DOI:** 10.1186/s12913-023-09968-6

**Published:** 2023-09-20

**Authors:** KL Bagot, E McInnes, R Mannion, RD McMullan, R Urwin, K Churruca, P Hibbert, JI Westbrook

**Affiliations:** 1 Nursing Research Institute –St Vincent’s Health Network Sydney, St Vincent’s Hospital Melbourne and Australian Catholic University, Fitzroy, VIC Australia; 2 School of Nursing, Midwifery and Paramedicine, Australian Catholic University, Fitzroy, VIC Australia; 3 School of Nursing, Midwifery and Paramedicine, Australian Catholic University, Melbourne, Australia; 4 Health Services Management Centre, University of Birmingham, Birmingham, UK; 5 Australian Institute of Health Innovation, Macquarie University, Sydney, Australia

**Keywords:** Unprofessional behaviours, Professionalism, Organizational Culture, Qualitative, Professional Accountability, Speaking up, Hospitals

## Abstract

**Background:**

The critical role that middle managers play in enacting organisational culture change designed to address unprofessional co-worker behaviours has gone largely unexplored. We aimed to explore middle managers’ perspectives on i) whether they speak up when they or their team members experience unprofessional behaviours (UBs); ii) how concerns are handled; iii) the outcomes; and iv) the role of a professional accountability culture change program (known as *Ethos*) in driving change.

**Methods:**

Qualitative, constructivist approach. Five metropolitan hospitals in Australia which had implemented *Ethos*. Purposive sampling was used to invite middle-level managers from medicine, nursing, and non-clinical support services. Semi-structured interviews conducted remotely. Inductive, reflexive thematic and descriptive thematic analyses undertaken using NVivo.

**Results:**

Thirty interviews (approximately 60 min; August 2020 to May 2021): Nursing (*n* = 12), Support Services (*n* = 10), and Medical (*n* = 8) staff, working in public (*n* = 18) and private (*n* = 12) hospitals. One-third (*n* = 10) had a formal role in *Ethos*.

All middle managers (hearers) had experienced the raising of UBs by their team (speakers). Themes representing reasons for ongoing UBs were: staying silent but active; history and hierarchy; and double-edged swords. The *Ethos* program was valued as a confidential, informal, non-punitive system but required improvements in profile and effectiveness. Participants described four response stages: i) determining if reports were genuine; ii) taking action depending on the speaker’s preference, behaviour factors (type, frequency, impact), if the person was known/unknown; iii) exploring for additional information; and iv) addressing either indirectly (e.g., change rosters) or directly (e.g., become a speaker).

**Conclusions:**

Addressing UBs requires an organisational-level approach beyond supporting staff to speak up, to include those hearing and addressing UBs. We propose a new hearer’s model that details middle managers’ processes after a concern is raised, identifying where action can be taken to minimise avoidant behaviours to improve hospital culture, staff and patient safety.

**Supplementary Information:**

The online version contains supplementary material available at 10.1186/s12913-023-09968-6.

## Background

Unprofessional behaviours (UBs) in healthcare (e.g., rudeness, humiliation, bullying, harassment, assault) [1–3] are associated with profound and damaging negative consequences [3] for individuals, organisations and patients [3–6]. Calls to address UBs in healthcare settings have long been reported [7, 8] yet they are a persistent global problem [3, 9]. A 2018 systematic review of 22 papers from the United States, Canada, Europe, the United Kingdom and Australasia, examining bullying, undermining behaviour and harassment within surgical settings identified consistently high prevalence rates (25–59%) [3]. A 2020 Australian survey revealed over 90% of 5178 hospital staff had experienced UB in the preceding year, with almost 40% reporting incivility or bullying behaviours on at least a weekly basis [9].

Improved care and staff outcomes are associated with positive organisational cultures which support staff to raise safety concerns, including UBs impacting the delivery of safe care [10]. As such, the importance of enabling ‘speaking up’ climates has increased and initiatives such as the UK’s “Freedom to speak up” guardian program [11], along with targeted staff training programs in ‘Speaking up’ are prominent [12–15]. These programs aim to address many of the recognised individual and contextual reasons why people do not speak up: fear of repercussions [16, 17], influence of hierarchy [18], personality [19] and the desire to maintain working relationships [7, 16].

Organisation-wide approaches are recommended [1, 2], yet high quality evidence-based interventions to address UBs are limited [2, 14, 20], with inconsistent results about effectiveness [14]. Professional accountability programs are promoted as one organisational approach to reduce UBs and have demonstrated promising effects [1, 2, 21]. These programs consist of a tiered process beginning with non-punitive, informal feedback delivered by peer messengers to hospital staff to raise their awareness of the effects of their behaviour [1, 22, 23]. Drawing on elements of a program at the Vanderbilt University Medical Centre in the United States [1, 22, 23], St Vincent’s Health Australia designed and implemented a whole-of-hospital professional accountability and culture change program called *Ethos *[24, 25]. *Ethos* includes staff training to build capacity in recognising and speaking up about UBs, and an online messaging system that allows staff to submit messages related to co-workers’ UBs. Submissions are assigned to peer messengers who then provide feedback to the subject of the submission during an informal conversation. The aim of *Ethos* is to increase awareness, provide opportunities for reflection and action prior to concerning behaviours potentially escalating, as well as facilitating the identification and recognition of positive staff behaviours. Since 2017, *Ethos* has been implemented in eight hospitals across three Australian states.

While senior organisational champions of such programs are crucial in supporting change, middle managers are tasked with enacting and supporting the program at an operational level. Little previous investigation of middle managers’ experiences with reporting and responding to reports about UBs has been undertaken. With an on-the-ground managerial function, middle managers are usually the first line responders for reports about UBs by staff that they supervise [26]. They are therefore able to provide insights about how to improve the reporting of and response to UBs.

### Current study aims

Within the context of a professional accountability program designed to reduce UBs [24], we interviewed middle managers about their experiences in dealing with UBs. We aimed to investigate hospital middle managers’ perspectives about:

i) whether they speak up to either more senior staff or to people committing UBs when they or their team members experience UBs;
ii) how they handle raised concerns;
iii) the outcomes of speaking up to those committing or reporting UBs; and
iv) the role and value of a professional accountability culture change program (known as *Ethos*) in addressing UBs.

## Methods

### Design and setting

This study was part of a larger, comprehensive mixed-methods evaluation of the *Ethos *program. We used a multi-site qualitative descriptive design, conducted in public and private hospital settings from St Vincent’s Health Australia based in two states. In Australia, there are both public (taxation-funded, government regulated) and private (two thirds privately funded with one third government funding) hospitals [27]. The Human Research Ethics Committee of St Vincent’s Hospital Melbourne approved the multisite study (HREC/17/SVHM/237). The study is presented in line with the COREQ guidelines [28].

### Intervention description

Details on the development, implementation and early results of the *Ethos *program have been reported elsewhere [21, 24, 25]. *Ethos* is a peer-led, early intervention professional accountability program available to all hospital staff (clinical and non-clinical). It targets behaviours requiring informal or low-level intervention (e.g., intimidating behaviour, derogatory remarks or jokes), and as such, augments, not replaces, existing disciplinary processes (e.g., serious complaints are referred to Human Resources; HR). *Ethos*has three arms: i) an all-staff capability building and training module for safe behaviour which includes training on how to ‘speak up’, ii) an anonymous online messaging system accessible to all staff to report both unprofessional and positive behaviours, and iii) a tiered accountability pathway where messages are triaged and allocated to trained peer messengers (to deliver ‘messages of reflection’ regarding unprofessional/negative behaviours) or to line managers (to deliver ‘messages of recognition’ regarding positive behaviours). Reflection messages are triaged and peer messengers have informal, confidential conversations with the subjects of submissions to increase awareness of their behaviours, and to encourage reflection, with no formal punitive consequences. Only when behaviours are persistent or identified as extreme (e.g., physical or sexual assault) are formal disciplinary processes commenced [24].

### Participant sampling and recruitment

Participating hospitals (*n* = 5) were all academic/teaching hospitals, with 22,605 to 62,998 annual admissions, 217 to 797 inpatient beds, and medical staff (e.g., physicians, surgeons) mostly employed as independent contractors and non-medical (e.g., nursing, allied health, support staff) staff directly employed by the hospital. All hospitals had implemented *Ethos* in the previous two years.

We used purposive sampling to invite middle managers from three public and two private hospitals. The Director of Nursing, Director of Medicine, Director of Acute Care Services and/or Director of Operations at each site were provided with a summary of the project, and were asked to nominate potential middle managers who met the inclusion criteria, including those who had a formal role within *Ethos* (e.g., peer messenger, member of Triage team), and those who did not. Inclusion criteria were medical, nursing or support services staff (e.g., those who worked within cleaning, food or other hospital non-clinical services) and who were:

1. middle managers who directly supervised staff (e.g., Nursing Unit Manager, Program manager, registrar, medical supervisor, support team manager); and
2. willing to participate in an individual interview conducted in English, focusing on UBs in their work area.

A researcher (EM) with no prior relationship with eligible personnel, emailed nominated middle managers an invitation to participate in interviews, providing a project summary and Participant Information Sheet. Those interested could make an interview appointment with the interviewer that suited them (implied consent) and active, verbal consent was sought at the commencement of each interview. Participation was voluntary, confidential, with no remuneration.

### Data collection procedures

One researcher, who was independent from the design, development and implementation of the project and the *Ethos* program, and not involved or affiliated with any of the hospitals (KB; female, PhD, Psychology, experienced qualitative health services researcher) conducted individual interviews remotely (using an on-line video platform) using a semi-structured interview schedule. The interview schedule (Figure S1) consisted of open-ended questions with prompts that covered four key areas reflecting the study aim: i) describing UBs within the organisation, ii) raising concerns by team members or themselves about UBs, iii) their responding to UBs, and iv) views on *Ethos*. Each section had a number of prompts to explore how UBs were addressed in each participant’s work team or area (e.g., ward, theatre, kitchen, etc.). The schedule was reviewed by the researchers and piloted on two individuals with clinical backgrounds but not involved in any of the hospitals or participating organisations. No amendments were made. To encourage speaking freely in each interview, the interviewer advised participants of their independence from both the hospital and *Ethos*, that all names and identifying details would be removed, and that they could review and edit their transcript prior to analysis. Interviews were recorded, professionally transcribed and transcripts were de-identified for analysis. Researcher field notes were taken throughout interviewing and analysis. There were no repeat interviews.

### Data analysis procedures

With a constructivist lens [29], analysis was undertaken inductively, following established procedures for reflexive thematic analysis [30]. First, one researcher (KB) undertook familiarisation with the data through iterative review of all transcripts (phase 1), conducted initial semantic coding from text analysis (phase 2), grouped and organised related codes (phase 3) and then identified and refined categories and sub-categories (phase 4). Key themes and sub-themes were identified (latent coding) and named (phase 5) with final themes and sub-themes endorsed by all authors (phase 6). Throughout analysis, interim results were reviewed, probed and discussed with EM (female, PhD, Nursing, experienced qualitative researcher), and presented to the wider research and investigator team for review and discussion. Interview data were compared and contrasted between professional groups, the public and private hospital sites, and between those who did and did not have a formal role in *Ethos*. A descriptive thematic analysis was undertaken to summarise managers’ processes and barriers and facilitators to using *Ethos *[31].

Analyses were initially undertaken with NVivo (v12) [32], with themes and sub-themes for phases 4–6 identified and finalised using a virtual whiteboard (www.ideaflip.com). Verbatim quotes corrected for grammar from each discipline illustrate themes/sub-themes.

## Results

### Participants

Thirty interviews of approximately 60 min duration were conducted between 13 August 2020 and 11 May 2021 (Table 1). There were 23 female and 7 male participants with management role experience ranging from less than 1 year to 14 years (Mean = 4.3 years, SD = 3.4). Ten participants had a formal *Ethos* role (e.g., *Ethos* messenger, triage team member). Six participants reviewed their transcript (three edited content).

**Table 1:** Participants by discipline and location, and formal involvement in the Ethos program

**Department**	**Total**	**Location**		**Formal Ethos role^**
		**Sydney**	**Melbourne**	
**Nursing**	12	9	3	4
**Support**	10	7	3	4
**Medical**	8	4	4	2
**Sub-Total**	**30**	**20**	**10**	**10**

Note: ^Formal Ethos roles included member of Ethos messaging Triage team, Peer messenger, or champion of Ethos

### Reporting and addressing unprofessional behaviour

Participants spoke of UBs they had experienced, witnessed or had been reported to them. Appendices summarise the types of UBs described (Table S1), between which staff groups (Table S2) and their impact (Table S3). Three key themes (‘staying silent but active’, ‘history and hierarchy’ and ‘double-edged swords’) with sub-themes (underlined in text) were identified which encapsulate factors relevant to people reporting and addressing UBs. Illustrative quotes by discipline are presented in Table 2.

**Table 2:** Themes, sub-themes and illustrative quotes relating to why people do not speak up and unprofessional behaviour continues

Theme	Sub-theme	Illustrative quotes		
		**Medical**	**Nursing**	**Support Services**
Staying silent, but active	Protection of self	“… because the training programs are so difficult to get onto … those tiny, tiny things make a difference. (027)	“I think there's a lot of people that don't go there with people … because they fear for their job.” (009)	“They're only on a year contract. You don't want to ruffle any feathers.” (011)
	Justify or excuse behaviour	“He just can't control his temper. … You can forgive people for trying their hardest.” (008)	“We see certain behaviours as … we package it up as them having a bad day, or that’s just part of their personality.” (005)	“I don’t think he can control it. … Again, not because he’s a bad person, but just the way he behaves.” (014)
	Emotional reframing	“… she'd just let loose at me in front of everyone in the office. So as soon as she left, they all turned around and were like, ‘You're all right. She's done this before. Don't worry about it.’ That made me feel better that it's not me. It's not me. It's fine.” (027)	“When I heard about that [surgeon being unprofessional towards a new nurse] I was really annoyed. But what I did was, I sent him an email and I said …’I just want to give you some really positive feedback’ … [Since then] he’s been really good. That was two weeks ago.” (017)	N/A – *Support Services’ Managers referred to addressing *via* policies rather than using emotional reframing of situation*
	Enable with workarounds	*N/A – Medical Middle Managers did not refer to workarounds*	(Nurses working with a known offender surgeon) *“*Once they’re pulled out and never have to do his list again, that makes them happy. So, that’s what I do … we roster so that it works out okay.” (017)	“We've recruited people that fit the team a little bit better and there's less of that friction that there was.” (015)
History and hierarchy	Individual, personal experiences	“I don't want to tell anyone because clearly everybody knows this is an issue and just puts up with it. … we just tolerate this behaviour here.” (028)	“The new grads don’t have that resilience yet. But it certainly has an impact. People don’t forget, that’s for sure.” (012)	“Pretty much nothing, just make some decision then after that pretty much stop there. There's no discipline action, no warning as to the issue, etcetera.” (019)
	Workplace, organisational culture	You're expected, as a junior doctor, to just deal with it and just—it's like a—what's the word—you've got to develop a thick skin. It's just expected I think.” (007)	“The problem is, these people make so much money for the hospital that—they’ll be spoken to about their behaviour time and time again, but the bottom line is the dollar.” (017)	“There's nothing we can do, that's the thing. This is a culture that's been here since I started. There's just really nothing you can do.” (015)
Double-edged swords	One person makes a big difference (could be a positive or negative impact)	“She just kind of screamed at me and run off. … Then that night I had to work with her again and I was kind of terrified to talk to her at all because she was so scary.” (028) “Our codirector organised that. It kind of blew my mind. Yeah. He’s like, we want to say what we can do to help … see if we can come up with some solutions. Which I thought was amazing.” (028)	“I know that she [manager] would do something—or she would address it with those people.” (012) “I haven’t muttered one word to him. So, he’s stayed away from me because he knows that he’s finally—he’s done it twice to me. He knows that he’s broken me.” (017)	*“*I have a very good direct report. My manager who I've had in and out of my career here, I know that she's available if there's any concerns, or if I need to bounce ideas off her.” (001) *“*So, she felt completely disrespected in the situation. My manager was frustrated with her because why didn’t we just provide it? It was just a sandwich. That's how everyone sees it; it's just a sandwich, just give it. But it was breaking the rules and my staff member didn’t feel comfortable doing that.” (015)
	Emotional management shifted from one person to another (manager retains issue once issue raised)	“Then I’m just kind of holding it and then I might talk to my boss or kind of keep my ear to the ground or maybe think about how I can raise it in a very de-identified way.” (010)	“Twice, two doctors that were ETHOSed [given an Ethos Reflection message] came to me asking who had ETHOSed them. … I wasn’t supposed to know, and I told them I didn’t know anything about it. It was awful.” (017)	“So normally what we do is we console them,” (018)
	Preference for directly addressing (but doing so takes a toll)	“Like that rude registrar, I wasn't going to go out of my way to pick up the phone and call them back and say, you're being really rude. I was a bit shellshocked afterwards.” (029)	“It [complaint] would have come to me and then I would have had to have spoken to the surgeon, who would have probably given me … excuses as to why he was behaving the way he was. Then I probably would have got upset with him because he'd be critical of the staff.” (016)	*“*So basically what I did, I spoke to the person thinking that that was the right thing to do, then it just tarnished me from then on.” (001)
	Confidential and anonymous system (one-sided; only works for reporting not addressing)	“It's really hard because you need the anonymity and at the same time that makes it really hard.” (028)	“You will understand it’s confidential and I won’t be able to tell you what’s been done.” (026)	“The person who puts in the Ethos, they don’t get any feedback, they don’t get anything.” (023)

### Staying silent but active

Middle managers who had experienced or were aware of incidents of UBs undertook additional cognitive, emotional or behavioural work to avoid having to address individuals committing UBs and to ensure optimal outcomes either for themselves (avoiding repercussions), others, or patients (availability of clinician to continue working).

Participants across all groups noted some individuals who experience UBs *stay silent but active;* that is, concerns are not raised but they engage in other cognitive, emotional or physical behaviours instead. They remain silent for protection of self against the perceived personal repercussions of speaking up. Actual or potential negative outcomes included: becoming known as a troublemaker which could influence acceptance to a clinical program (medicine); not being allocated to a desired surgical list (nursing); or losing a preferred shift day/time (support services). For some, the unprofessional behaviour was justified or excused due to the stressful environment (i.e., not the fault of the individual committing the unprofessional behaviour), the patient case involved (i.e., dangerous case and person committing unprofessional behaviour really cares about patient), the personality (i.e., cannot be changed) or contribution to the hospital (e.g., surgeons attract money) was accorded more importance than the individual subjected to unprofessional behaviour (e.g., nurse being yelled at by surgeon). Emotional reframing occurred with participant 008 interpreting the unprofessional behaviour of a known repeat offender as demonstrative of caring for their patients and said “I love him”. Another described a doctor who did not gown or glove for a patient during the COVID-19 pandemic as “they’re naughty” rather than someone behaving unprofessionally (Participant 001). Some participants implemented workarounds to enable care to continue without having to directly address the UBs (e.g., schedules rearranged ensuring individual committing the unprofessional behaviour and recipient/s were not working together).

### History of Inaction and hierarchy

The theme *history and hierarchy* of UBs captures the iterative process of individual, personal experiences and workplace, organisational culture. Many participants referred to the influence of personal experiences and how the organisation’s approach to addressing UB contributed to their perception of workplace culture, particularly in relation to the acceptance of UBs. For some, there were references to a personal history of speaking up or reporting UBs with no further action taken. There were also examples of participants being aware of ongoing UBs by known repeat offenders, even after reports had been made. Over time, this lack of action led to perceptions of a workplace culture that certain behaviours by certain people (known offenders) were accepted. Many participants also spoke of the role of hierarchy in inhibiting speaking up or addressing UBs. For most, the person committing UBs was more senior, held more power or expertise and as such, any UB committed was less likely to be reported or addressed. Inaction for these experiences also contributed to a workplace culture whereby UBs were accepted if committed by certain people. Where there were established personal or professional relationships, these could limit the ability to directly address UBs. When using the *Ethos* reporting system, participants had mixed opinions whether there had been improvements in behaviour. Some middle managers reported that *Ethos* alerted them to ongoing situations that would otherwise have remained hidden.

### Double-edged swords

Factors involved in experiencing or addressing UBs had both positive and negative consequences: *double-edged swords*. Participants described circumstances where one person makes a big difference (could be a positive or negative impact) to an individual’s experience of UBs. Having a person to discuss a concern was of great importance (either team members approaching middle managers or middle managers in turn approaching someone), diluting the negative impact of experiencing UBs. All participants indicated that they had someone with whom they could debrief, and that they worked at ensuring they were approachable to members of their team. However, individuals committing even a single, isolated unprofessional behaviour could have a significant and potentially long-term negative impact on a recipient.

Middle managers being approachable for team members to raise issues related to UBs led to emotional management being re-allocated from one person to another. Managers accepted an additional burden in managing the emotional impact for the recipient, and also the impact on themselves. Some participants noted that individuals experiencing UBs wanted to vent and to be heard but wanted no action to be taken. However, this led to participants having to care for upset team members while ensuring patient care. Participants carried the stories and experiences of others, and in some cases with no outlet or prospect for change. Similar comments were raised by *Ethos* messengers, as they knew something about staff in their workplace that they would normally not know. Managers enjoyed delivering positive *Ethos* reports (messages of recognition) which were well received. Some suggested these be provided in the form of a certificate or be incorporated into organisational procedures (e.g., included in personnel file).

Most participants noted a preference for directly addressing the person who committed UBs witnessed or reported to them in their role as a manager (outside of any role as an *Ethos* peer messenger), but doing so took a cognitive, emotional and physical toll. Addressing behaviour required consideration of timing and content of conversations, drawing on personal strength and confidence. At times, this included dealing with responses from the individual who had committed UBs whose response could also be unprofessional (e.g., sarcastic, angry, aggressive) or the repetition of some offenders. Many participants who were also *Ethos* peer messengers mentioned the benefits of receiving *Ethos* messenger training in how to conduct difficult conversations with others in the workplace, proving useful in their managerial role by providing phrases and strategies when dealing with their own team members. Participants indicated that they would only hesitate in addressing UBs directly with the person committing the behaviours if recipients had requested it not be addressed, or if patient safety was an immediate priority (e.g., managers witnessing UBs would ensure clinical care for patients was completed prior to speaking with the staff member exhibiting UBs).

Often when discussing how to address UBs, participants noted the importance of a confidential and anonymous system/process for reporting UBs. However, this was also viewed as a negative, as once *Ethos* messages are submitted to the online system, those reporting are not provided with any follow up information (e.g., if the behaviour was being addressed, if the individual committing the unprofessional behaviour had been spoken to or had committed to changing their behaviour).

### Group differences within themes

There were no discernible differences by geographic location or public/private hospitals.

Non-clinical support services participants referred to documented policies and processes to deal with reports of UBs, while medical and nursing groups provided examples of emotional reframing. Nursing and support services’ middle managers referred to work arounds, while medical middle managers did not. All groups generally followed the same processes of assessing and addressing UBs (see next section). There were also no group differences in perceptions of the *Ethos* online messaging system. It was identified as having value: a system beneficial to those without the confidence to speak up directly; a structure and process to follow with non-punitive outcomes; and line managers of those reported as committing UBs did not have to be involved in addressing unprofessional reports (as messages were conveyed by trained peer-messengers). However, some were not sure if the reporting system was still available, or could not recall how to access or use it. Further barriers and facilitators of the *Ethos* program are outlined in Table 3.

**Table 3:** Facilitators and Barriers to using the Ethos Program

Theme	Facilitators	Barriers
**Individual**	Anonymous	Concerned they are identifiable
	System for those not confident speaking up directly	Sometimes do not know required details (e.g., name of perpetrator)
	Others can submit messages	Low awareness of how it works
	Removes work for the manager	Prefer to speak directly to perpetrator
**System**	Removes power imbalance	Technology / intra-net based, forget how to access, password
	Provides structure and processes	No right of reply, one-sided
	Informal, non-punitive	Don’t know if action is taken or not
	Peer, not colleague or junior, provides feedback	Mocked, used as a threat
**Effectiveness**	Identified hidden issues for managers	Behaviour has not improved
	Perpetrators not realising impact until received message, behaviour improved	Outcome not guaranteed
	Training in how to conduct difficult conversations	Management behaviour, not unprofessional behaviour
	Positive messages have a strong positive impact on recipients	-

### Stages undertaken by middle managers hearing about UBs (aims ii and iii): A model of how hearer’s approach UB reports

**Fig. 1:**
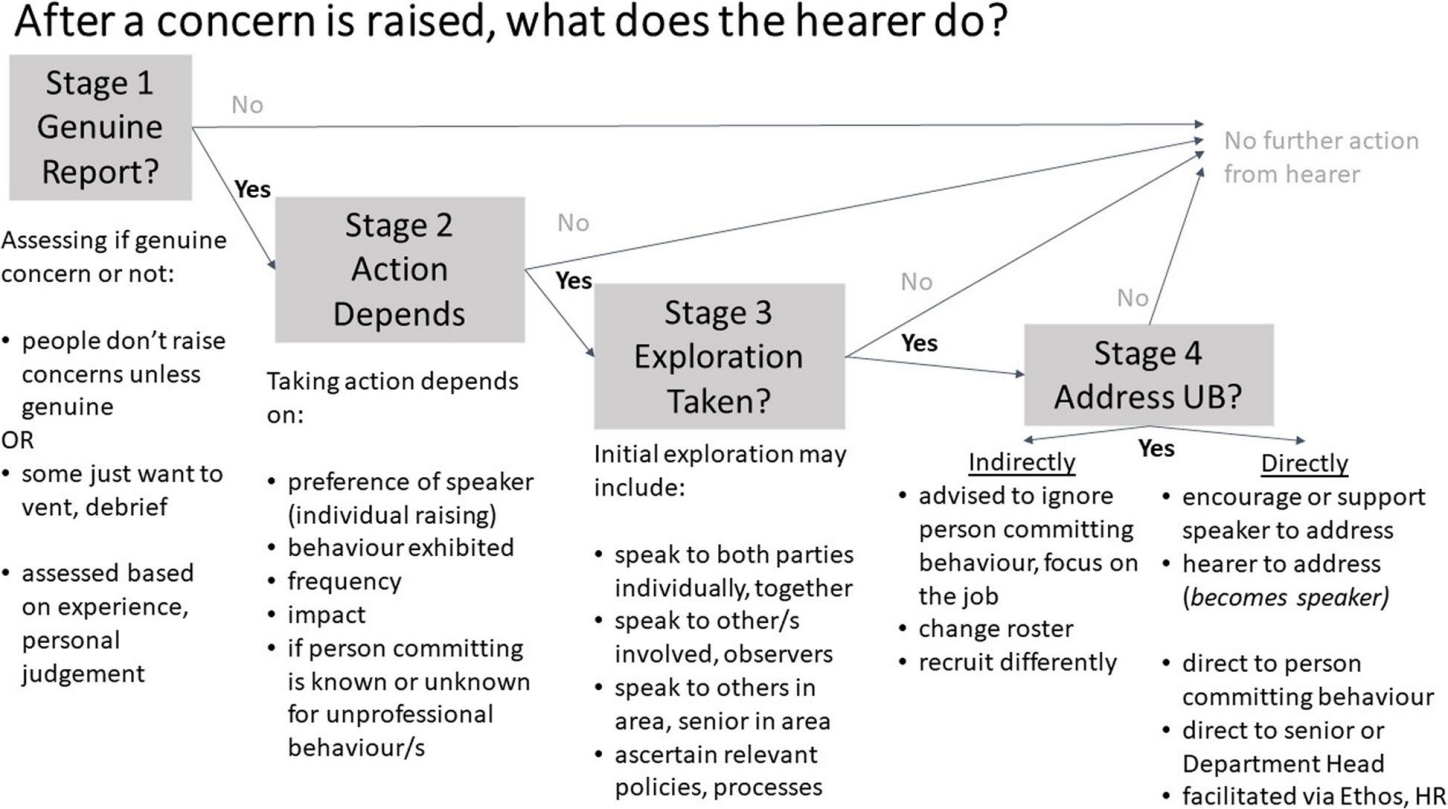
Hearer’s model outlining processes middle-level managers go through when hearing and addressing unprofessional behaviours

The themes presented above are influential in reporting or addressing UBs. The following describes the stages undertaken by managers when staff members report UBs (speakers) to them (hearers). When considering if and how to address UBs, the four stages of hearers’ processes (Fig. 1) are:

Stage 1) GENUINE REPORT—determining whether a concern is genuine or not. While some middle managers indicated that concerns are not raised unless they were genuine (therefore all concerns raised are genuine and require consideration of actions to address the concern), others acknowledged that some staff merely want to vent or debrief about a situation that may or may not reflect an unprofessional behaviour or may not be a genuine concern, and therefore not have any specific action subsequently taken. Middle managers indicated they determined if content of discussion was of genuine concern based on the details provided, factors such as their experience, their personal judgement and speaking with others.
Stage 2) ACTION DEPENDS – even if identified as genuine, taking action depended on a range of factors, including: the recipient’s wishes or preference, the type and extent of behaviour, the frequency of behaviour, the impact of behaviour, and if the individual committing the unprofessional behaviour has a known history of UBs;
Stage 3) EXPLORATION TAKEN—depending on factors in stages 1 and 2, the manager may take initial action and seek additional information by informally speaking to others involved, individually or together, speak to others in the area and/or refer to formal policies and processes. They may also seek input from peers or their managers to assist with understanding the situation and options for addressing; and
Stage 4) ADDRESS UBs—depending on the combination of factors in stages 1, 2 and 3, managers may take no further action (e.g., if not genuine, if recipient requests), address indirectly (e.g., advising staff member raising concern to focus on the job or avoid co-worker committing UBs, recruiting differently, changing roster, etc.) or address directly (e.g., encourage or support person raising concern to address UBs themselves or the hearer addresses, either direct with person committing UBs or involving their senior/Department Head or to work within organisational systems such as via the *Ethos* Program or HR).

## Discussion

Reducing UBs in healthcare settings is of global interest [3]. Our results indicate that despite long-held awareness of their detrimental impacts, UBs continue to occur in contemporary healthcare settings, among clinical and non-clinical personnel. While some middle managers noted improvements in how UBs were being addressed in their workplace (e.g., support from senior management, policy rules in place), further opportunities were identified, placing middle managers hearing UBs as pivotal to addressing them.

### Supporting speaking up necessary but insufficient for directly addressing UBs

Middle managers indicated they would generally not hesitate in addressing UBs if a team member reported it or if they experienced it themselves. However, in addition to directly addressing (e.g., with person exhibiting UBs or reporting system or via HR), responses included taking no action (e.g., at recipient/speaker’s request, debrief only), delaying action (e.g., when patient care was prioritised), and addressing indirectly (e.g., change in roster or recruitment). Decisions not to address UBs highlighted the influence of hierarchy (e.g., more senior personnel committing UB), financial contribution (e.g., surgeons bring in money) or clinical expertise (e.g., specialist skills and knowledge) [33] illustrating recipients’ perceived lower power [34]. Confidential systems like *Ethos*can provide avenues to raise concerns anonymously and informally [35], with third parties (*Ethos* Triage Team) reviewing submissions and identifying ongoing patterns.

Some managers justified, excused or positively interpreted those committing UBs to perceive them favourably (cf., Stockholm Syndrome) [36], precluding managers from having to directly address them. A previous study found that despite attending physicians’ claims that they would speak up in the face of UBs, in 160 h of observations, no direct conversations were witnessed, and non-verbal or indirect verbal responses were not detected by intended recipients [7]. Concepts such as the ‘deaf effect’ (i.e., not hearing raised concerns/complaints) [37], ‘hearer courage’ [38] or different management styles [39] suggest hearers’ responses vary [39], impacting if UBs are addressed. Middle managers may believe they are identifying, acting on or addressing UBs, but those who experience unprofessional behaviour may have different views. If speaking up is encouraged but direct action is not taken, speaking up culture is not maintained [40].

With a focus on speaking up, individuals experiencing UBs shoulder the initial responsibility of addressing UBs; that is, they need to raise the concern, even where individuals known for persistent UBs are already identified. Managers have additional workloads involving cognitive, emotional or behavioural actions in comforting upset team members, planning or strategising approaches to consider and implement, through to managing their own responses. While mandatory reporting guidelines cover serious UBs [41], for other UBs some participants had not received any training to address reports of UBs.

Promisingly, some participants reported positive outcomes from submitting *Ethos* reports, including improvements in an individual’s behaviour, and awareness of hidden UBs. Others, however, indicated no changes in UBs or were unsure if any action had been taken (either by HR or the *Ethos*team, as relevant). Lack of feedback has previously been reported as a barrier to patient safety incident reporting [42]. Feedback could include reporting actions back to individuals submitting concerns or communicating the online messaging system metrics (e.g., submission numbers) to normalise and encourage its use. Providing positive feedback within programs (as with *Ethos*) is also an important element supporting improvements in organisational culture and outcomes[43, 44].

### What happens after speaking up? A model of hearers’ responses

Previous models have focused on those experiencing UBs; that is, speakers’ processes (e.g., voice, silence, voiceable concern) [45, 46]. There is a dearth of literature on what hearers do when witnesses or targets of UBs raise concerns (i.e., speakers). Our work provides a four-stage model (Fig. 1) specifically detailing the sequence of hearers’ actions when UBs are raised. Similar to the first two stages of our hearer’s model (Stage 1: Genuine Report?, Stage 2: Action Depends), previous work examining acts of wrongdoing [37] (or UBs in our context) identified six intrinsic dimensions are used to assess the behaviour: i) whether the determination is subjective or objective, ii) whether it is based on values or facts, iii) frequency of behaviour, iv) was it intentional or not, v) is there a public interest dimension, and vi) if the recipient of the behaviour is vulnerable. Our participants reported using similar assessments when determining if a concern is genuine (e.g., subjective or objective, values or facts) and referenced unprofessional behaviour frequency as a motivator for action. Mannion and colleagues [47] suggest that the public interest criterion is always met for UBs in healthcare settings, and patients are considered vulnerable. Staff may also be considered vulnerable, given reasons provided for not speaking up (e.g., fear of repercussions) [16, 17]. Notably, these factors could apply to both parties: the witness or target raising the concern (speaker) and then the hearer to address. For example, the speaker literature [35] includes the informal exploratory processes such as sense-checking or fact finding undertaken by those with a potential concern. Similarly, our managers reported an exploration phase.

The model we present extends approaches with Stage 3: Initial Exploration, and in Stage 4: Addressing UBs distinguishing between two approaches middle managers undertake: indirectly or directly addressing reports about unprofessional behaviour. Prior research suggests that indirect or avoidant approaches are not successful strategies [48, 49], and yet were often reported by our participants. Our model identifies specific skills and actions to target in training and systems to support hearers of UBs. In turn, while the person receiving the information about UBs is a hearer, they may need to become a speaker for the behaviour to be addressed; Mannion et al. refer to this as recursive action [47]. Following the experiences of those involved in reported UB events is warranted to explore if and how individuals move between the hearer and speaker roles. Future research could explore the impact (e.g., emotional, cognitive, etc.) on middle managers’ hearing and potentially addressing UBs. We provide other specific recommendations (Table 4), based on our results, for improving how UBs are addressed in healthcare settings for the benefit of patient and staff safety [37].

**Table 4:** Recommendations for improving how unprofessional behaviour is addressed in healthcare settings

Target	Focus	Example
**Ethos – professional accountability culture change program**	Initiate and maintain awareness of Ethos program	Needs refresher campaign—increase awareness overall and specific processes to submit and what happens after submitted
		Provide updates of messages received and acted upon; provide certificates for Recognitions, formalise Recognitions in personnel file
	Consider tailored adaptations by Ethos program role	Submitters – Advised that their report has been reviewed or actioned Messengers—Selected to deliver messages to those easily accessible (consider same campus, on similar roster); real life examples used in training
**General**	Improve working relationships further to support addressing behaviours	Enhance inter-disciplinary relationships and communication
		Link relevant disciplines so patient journey, practices and policies clear
		Acknowledge and emphasise that all roles make a contribution to patient safety, regardless of discipline or hierarchy
	Training in conflict management and difficult conversations for all	Train in initiating, conducting and being in difficult conversations; responding to conflict situations; giving and receiving difficult feedback; raising, hearing and addressing unprofessional behaviour, including handling any personal impacts
		Self-management of emotions (e.g., frustration leading to incivility, responses to being advised of having exhibited UB)
	Systems in place to support all involved	Provide equal support and structure for both the speaker and the hearer of unprofessional behaviour reports. For example, training programs to support actions for those ‘speaking up’ as well as for those ‘hearing’, including identifying genuine concerns and addressing directly

### Limitations

All sites were teaching hospitals and participants were mostly female (reflecting the healthcare workforce), did not include representatives from all professional groups, and potential participants were identified by senior management. Participants did not raise severe examples of UBs (e.g., sexual assault)[9], however *Ethos*is not designed to address severe behaviours and may have precluded participants raising such behaviours. Importantly, our model is based on middle management perspectives as hearers, and does not incorporate the perspectives of those reporting UBs (speakers). Future research is required to evaluate the model’s applicability by those speaking up. The study was conducted during the COVID-19 pandemic exacerbating recruitment difficulties. However, COVID was not a primary source for UBs in this study’s settings[50]. Finally, we did not include all hospitals using the *Ethos* program, which had been established for varying periods across the participating hospitals (2–3 years).

## Conclusion

Our work presents the first model of hearer’s actions when individuals speak up to middle managers about UBs. These four stages provide key decision points that organisations can use to support managers in addressing UBs. Professional accountability programs like *Ethos* play an important role, including addressing underlying individual and organisational reasons for UBs through awareness and training, and implementing procedures and systems supporting the reporting of UBs. Systems for change must extend beyond speaking up to include training and systems supporting hearer actions, and consideration of the socio-political and psychological factors which influence hospital cultures.

### Supplementary Information

**Additional file 1. **

## Data Availability

The datasets generated and/or analysed during the current study are not publicly available due to ethical requirements but are available from the corresponding author on reasonable request.

## Abbreviation

UB: Unprofessional behaviour
