# Supplementary material for: Middle manager responses to hospital co-workers’ unprofessional behaviours within the context of a professional accountability culture change program: a qualitative analysis
Source: BMC Health Serv Res. 2023 Sep 20;23:1012. doi: 10.1186/s12913-023-09968-6 (PMC10510259; doi:10.1186/s12913-023-09968-6)
Supplement: Supplementary file 1 — Additional file 1. [file 12913_2023_9968_MOESM1_ESM.docx]

**Supplementals for manuscript: Middle manager responses to hospital co-workers’ unprofessional behaviours within the context of a professional accountability culture change programme: a qualitative analysis**

Bagot KL^1^, McInnes E^1,2^, Mannion R^3^, McMullan RD^4^, Urwin R^4^, Churruca K^4^, Hibbert P^4^, Westbrook JI^4*^

*Corresponding Author

Professor Johanna I. Westbrook

Director

Centre for Health Systems and Safety Research

Australian Institute of Health Innovation

Faculty of Medicine and Health Sciences

Macquarie University

^1^ Nursing Research Institute, St Vincent’s Health Network Sydney, St Vincent’s Hospital Melbourne; and Australian Catholic University

^2^ School of Nursing, Midwifery and Paramedicine, Australian Catholic University

^3^ Health Services Management Centre, University of Birmingham

^4^ Australian Institute of Health Innovation, Macquarie University

**Supplemental Figure S1 – Interview Schedule**

***Ethos* Middle Managers Interview Guide**

*Note: Questions were not always asked in this order to maintain a fluid momentum to interview*

1. **Raising concerns about unprofessional behaviour**
   1. Do you feel that as a staff member you have the resources and skills that you need to raise concerns about unprofessional behaviours?
   2. As a staff member, do you have confidence that your manager will respond appropriately and promptly to concerns about reports of unprofessional behaviours?

*Ask for reasons/examples*

- 1. Which groups in the hospital are most likely to be heard and responded to appropriately when they speak up about unprofessional behaviour?

*Prompts professional group/job, seniority,* *gender, age, cultural group*

- 1. Which groups in the hospital are least likely to be heard and responded to appropriately?

*Prompts professional group/job, gender, cultural group*

- 1. Overall, what do you think are the factors that enable or support you speaking up about unprofessional behaviours?
  2. Overall, what do you think are the factors that impede or stop you from speaking up about unprofessional behaviours?

1. **Responding to unprofessional behaviour**
   1. As a manager, if a member of your team raises a concern, would you feel adequately prepared/empowered in terms of how to respond/your ability to respond, and what action to take? Could you give me an example?

*Ask for examples – what was the issue, who raised the concern, what actions were initiated to address concerns, support given to person responding?*

- 1. How would you assess whether a raised concern is genuine or not?
  2. Can you provide an example of where you've responded to concerns well? What did you do?
  3. Are there any examples that you want to share that you could have facilitated a more effective response? What would have helped you?
  4. Overall, what are the factors that impede or stop you in responding appropriately to concerns about unprofessional behaviours?
  5. Overall, what are the factors that enable or support you in responding appropriately to concerns about unprofessional behaviours?

1. **Using the Ethos program to address unprofessional behaviour**
   1. Do you think people who work in your area/team have an understanding of what the ETHOS program is all about?

Can you tell me a bit more about why you think that?

- 1. Do you think ETHOS has helped people to speak up about unprofessional behaviours?

*Probe: Any differences noted in incidence of speaking up since ETHOS implemented.*

*Ask for examples; Probe what was the issue, who raised the concern, what actions were initiated to address concerns; other processes /strategies additional to ETHOS used*

- 1. As a manager, has the Ethos program helped you to identify unprofessional behaviour happening in your team/work area **that you were previously unaware of?**
  2. Has the Ethos program helped you to respond to/address unprofessional behaviour in your team?
  3. Has the Ethos system improved upon previous processes for dealing with unprofessional behaviours?

*Probe reasons for response and ask for examples*

- 1. Overall, is there a value to ETHOS and if so, what might that be? To you as an individual, your team, the hospital?

*Probe: Improved culture*? *Feelings of safety?* *Patient safety?*

- 1. Do you think ETHOS is sustainable in the long term? What do you think will ensure that ETHOS continues to be used? What would need to happen?

*OR If earlier questions indicate low visibility of Ethos* - What needs to happen for it to be used??
*Probe: Promotion? Training?*

- 1. What recommendations do you have for other hospitals implementing similar programs?

1. **Final thoughts and comments**

Is there anything else that you would like to add?

**Supplemental Table S1: Types of unprofessional behaviours raised and illustrative quotes**

|  | **Illustrative quotes** | | |
| --- | --- | --- | --- |
| **Type of unprofessional behaviour** | **Medical** | **Nursing** | **Support** |
| **Communication** |  |  |  |
| How people say things   - No respect, dismissive - Yelling, barking, shouting, hissing, spitting, violent, aggressive | *“In the middle of the COVID pandemic, some surgical registrar come up and stomped into the department and yelled, this far away from my face for about 10 minutes.” (#030, Medical, no formal Ethos involvement)*  *“Being rude on the phone when a more junior doctor has called a registrar for advice or for a consult. So, I think that’s a really common pattern.” (#010, Medical, no formal Ethos involvement)* | *”Probably the main thing that we experience is a lot of raising their voice because the nurse is not understanding what they want immediately, and that may be due to inexperience. So raising the voice is one major thing that we do see a lot. See a bit of eyerolling and a bit of grunting and groaning, and body language, I think is also one that is seen quite commonly.” (#021, Nursing, involved in Ethos)* | *“I do have staff that are yelling at me and almost pretty much spat at me before” (#019: Support, no formal Ethos involvement)*  *“Sometimes on the telephone, if the nursing staff are frustrated about a situation, they can be quite abrupt on the telephone, or demanding. We've even had nursing staff hang up on us on the phone, those sorts of things.” (#015, Support Services, no formal Ethos involvement)* |
| What people say (or don’t say)   - No communication, ignoring people - Demeaning or attacking comments - Name calling - Speaking in a language other than English | *“He looked at me and he said, ‘I want somebody who actually knows what they're doing to help’.” (#028: Medical, no formal Ethos involvement)* | *“Instead of using nicer ways of asking people to do things, it's more of a command which isn't appropriate either.” (#022; Nursing, no formal Ethos involvement)* | *“So when we have someone cleaning, they [doctors on rounds] will just tell them rudely– they don't say, please go out of the room, that would be much better. But they say just, get out, or small things like that.” (#018, Support Services, no formal Ethos involvement)* |
| **Work Performance** |  |  |  |
| Not doing their role | “*My member of staff explained to her that, ‘yes, it [an assessment test] was worthwhile doing’. Then this senior junior doctor had an argument with her, [saying] ‘it's pointless’ and carried on, and was really obstructive to repeating the test.” (#008: Medical, Ethos involvement)* | *“So, a team of three people with this person involved it would mean that the other two people had to pick up more than their fair share of work because she wasn't able to keep up at the same pace and adapt to the new computer programs. Because a lot of that - we've had a lot of change over the last 12 months with computers in our facility. So, new programs; every time you turn around, there's a new software program for something so she struggled - she was struggling with that. Then her response was just not do it and then someone else would have to pick up that because it hadn't - the job hadn't got done.” (#016, Nursing, no formal involvement)* | *“But the one they normally, for my encounter about people are angry at me it's actually more about their quality of their work. Such as for the hospital we actually need to make extra clean … we can't have dust floating around for some high-risk area.” (#019, Support Services, no formal Ethos involvement)* |
| Not respecting other disciplines | *“I've seen mostly the males - it sounds terrible - the male consultants or registrars. Just a few, not all of them will be like, that's a nurse’s job. I'm not doing that. I'm a doctor or I'm a doctor so my decision is the right one. You can't question me.” (#028, Medical, no formal Ethos involvement)* | *“But there are cases where the staff member is more than capable of doing it, and then there’s still that behaviour from the VMO because they just don’t want - it’s just taking then their time, because they might have to go a bit slower or they might actually have to teach.” (#017, Nursing, no formal Ethos involvement)* | *“There used to be six to eight doctors doing a ward round together and they just burst into a patient's room. So, you're in there already, and then sometimes you can't even get out because they've blocked the door and stuff like that. My staff have a computer on wheels to push around. So, trying to get that out of the room when they're trapped in a room by all these groups of people is also very awkward. I think the patient sometimes feels for us, we feel awkward because we obviously don’t want to be in there in a private conversation, but then sometimes a doctor is like, no, wait, just stop there, we'll only be two minutes. But that's not giving the patient their privacy as well.” (#015, Support Services, no formal Ethos involvement)* |
| Behaviours outside policy | *“So, he gave me his number - now this is an issue because if you send a patient’s photos to some complete random, then that random person has their photos and their details. Like you try and crop it out so there's no details but then also ask you for the Bradma [label with patient’s details] so they can look it up later. So, I was like, okay, can I just double check your number? Is it 04 - and then he hung up on me. I was like, should I just call him back or should I just send him the photos? But it was such an unpleasant encounter that I just sent the photos. Then 15 minutes later, of course I cried. It was 3:15 in the morning. I am like, do I wait, do I call him back, did I just send that to a random because if I sent it to a random, I'll never hear back from him.” (#028, Medical, no formal Ethos involvement)* | *“We're in a pandemic. She wasn't taking off her PPE in the right order. When corrected on it, she got quite angry and defensive to the nurse in charge.” (#003: Nursing, Ethos involvement)* | *“Well, I had one manager come to me regarding a VMOs poor performance and it was to do actually with COVID and not sort of - a potential COVID case, so was being triaged because they had a temperature. So the process would have been to gown and glove prior to going in to seeing the patient. The team had performed everything correctly in what they were doing, but the doctor at the time just walked straight in to the patient, didn't gown or glove, da-da-da.” (#001, Support Services, no formal Ethos involvement)* |

**Supplemental Table S2: Group dynamics of unprofessional behaviours and illustrative quotes**

| **Theme** | **Illustrative quotes** | | |
| --- | --- | --- | --- |
|  | **Medical** | **Nursing** | **Support Services** |
| Hierarchy  (role, discipline) | *“Sometimes it might be senior nurses upsetting junior doctors or vice versa.” (#010: Medical, Ethos involvement)* | *“The doctor’s orders are the correct orders and you just follow the directions from the doctor, having the more experience and the more knowledge.” (#016: Nursing, no formal Ethos involvement)*  *“[Consultants are] very high on the unprofessional behaviour delivery.” (#002, Nursing, Ethos involvement)* | *“So, from the receiving end, I can only talk about my team, which is cleaning and the food services, which is sort of at the bottom of the food chain.” (#022, Support Services, formal Ethos involvement)*  *“I would say that cleaners are looked at last compared to anyone above us.” (#018, Support Services, no formal Ethos involvement)* |
| Discipline-specific | *“it's either, you know, there's - Radiology who's been completely inappropriate in their interpersonal [communication] - or a surgical team that has a pattern of behaviour of being unnecessarily aggressive. Or anesthetic teams doing weird passive-aggressive things.” (#030: Medical, no formal Ethos involvement)* | *“We are in a private hospital, and regardless, it is still a teaching hospital, because obviously we take new grads on, and so there is - we have history in this hospital of poor behaviour, because it’s always been allowed, and you could say that a lot of our surgeons, not all, but a lot of the older ones are certainly spoilt in their behaviours.” (#017, Nursing, no formal Ethos involvement)* | *“I would say the majority of ETHOS we get are from the nursing staff, but I wouldn't say that has anything to do with the culture of the nursing staff. I think it's just that that's the biggest cohort of people.” (#011, Support Services, no formal Ethos involvement)* |
| Demographics (gender, age, cultural group/ethnicity) | *“But the impression that I get from them [nurses] is that they generally find female doctors harder to get along with than male.” (#028: Medical, no formal Ethos involvement)* | *“… sometimes it’s actually just their culture and how they come across. I think that gets a bit grey sometimes.” (#002: Nursing, Ethos involvement)* | *“Maybe actually some of the male doctors are probably less tolerant of us. But then in saying that, some are really good as well. I wouldn’t want to brand that it was always the male doctors.” (#015, Support Services, no formal Ethos involvement)* |
| Known offenders | *“He's a very senior surgeon within the hospital and a leader at a much higher executive level. He gets incredibly passionate about what he does, and he's in incredibly passionate about his patients, and he really cares about his patients. He works very, very hard, long hours, and he is definitely driven by doing the right thing for everybody. He's a hot headed, shouty, screaming, swearing person.” (#008: Medical, Ethos involvement)* | *“Everybody here in this department is aware of it and so is the whole organisation, so it's not a secret.” (#009: Nursing, no Ethos involvement)* | *“We actually haven't been surprised with really the majority of our messages that come through.” (#011: Support Services, Ethos involvement)* |

**Supplemental Table S3: Impacts of unprofessional behaviours and illustrative quotes**

| **Theme** | **Illustrative quotes** | | |
| --- | --- | --- | --- |
|  | **Medical** | **Nursing** | **Support** |
| Individual | *“Sometimes even a senior doctor is left in tears because the person on the other end of the phone is so rude.” (#010: Medical, Ethos involvement)*  *“I'm sure there are plenty of junior doctors who've been through this place on his team who just are never the same afterwards.” (#008; Medical, no formal Ethos involvement)* | *“There is one surgeon in particular - now, every six months, he breaks me.” (#017: Nursing, no formal Ethos involvement)* | *“[Unprofessional behaviour] makes them feel like they're not valued and listened to.” (#004: Support Services, Ethos involvement)*  *“So, she felt completely disrespected in the situation. My manager was frustrated with her because why didn’t we just provide it? It was just a sandwich. That's how everyone sees it; it's just a sandwich, just give it. But it was breaking the rules and my staff member didn’t feel comfortable doing that.” (#015, Support Services, no formal Ethos involvement)* |
| Organisational | *“It might seem like a small situation [having to support someone upset]. But it can have a big impact on how things run.” (#007: Medical, no formal Ethos involvement)*  *“Because you're going to have somebody in your room, you do really big interesting cases with lots of learning opportunities for everybody, so there are often people in the room who don't know you personally, who aren't accustomed to your passionate outcries. They are affected by it, and it puts them off working in the environment.” (#029, Medical, no formal Ethos involvement)* | *“We actually have no working relationship other than via email and it's not pleasant. It's really unpleasant.” (#009: Nursing, no Ethos involvement)*  *“Sometimes you see it by the flow of staff who just come and go, and you don’t know what that’s about. Is the turnover because the unit develops people really quickly and then they fly? Or do people leave because it’s awful?” (#026, Nursing, Ethos involvement)* | *“Well, it was detrimental to the organisation as well, from an organisational point of view. If you haven't got happy staff, they're not going to be doing their job. They're a crucial part of the organisation so - yeah, I mean I think it flowed down to everybody. You know what it's like, people get to hear and suddenly you start to need people transferred from a different department to work in there, well people don't want to go because they've heard about the behaviour that is in that group. So, yeah, I think it filters out to everybody.” (#011, Support Services, Ethos involvement)* |
| Patients | *“So he [consultant] wants to send the patient home but I don’t know if the patient has a broken bone. Do they need it plastered? It was just not the fracture that I was worried about. I don’t actually have any of these answers. I didn’t want to call him back.” (#028: Medical, no formal Ethos involvement)*  *“This big kind of mythology starts to come up. So, for example one of the issues I have in my role clinically is junior doctors not escalating for help in the middle of the night. Of course, if there’s this big mythology in the hospital that every senior doctor is going to yell at you if you call, of course they’re not going to call. In actual fact that’s not the truth. But then these stories just get bigger, then everyone hears about them.” (#010, Medical, Ethos involvement)* | *“They [nurses] get worried about escalating. They don’t want to hassle the doctor. They leave it until the very last minute where maybe it’s too late and we could have done something.” (#002: Nursing, Ethos involvement)*  *“Yeah it has so many effects, even to the patients. The patients sometimes say ‘That doctor’s really aggressive. She shouldn’t speak to you like that.’ You're hearing those types of things going on. Some patients are very astute in our area, because they sit up quite a lot, they're walking around, they're mobile, they maybe over hear conversations, they catch snippets out the door. For sure they pick up on those things.” (#005, Nursing, no formal Ethos involvement)* | “*I'm hearing it a little bit too much of things being said in front of patients. … It's really demeaning, it's that inappropriate behaviour towards patients. It's so uncomfortable for a patient to be listening to that, as well as unprofessional.” (#004, Support Services, Ethos involvement)* |
